# Hepatitis E Virus Occurrence in Pigs Slaughtered in Italy

**DOI:** 10.3390/ani11020277

**Published:** 2021-01-22

**Authors:** Eleonora Chelli, Elisabetta Suffredini, Paola De Santis, Dario De Medici, Santina Di Bella, Stefania D’Amato, Francesca Gucciardi, Annalisa Guercio, Fabio Ostanello, Vitantonio Perrone, Giuseppa Purpari, Gaia Sofia Scavia, Pietro Schembri, Bianca Maria Varcasia, Ilaria Di Bartolo

**Affiliations:** 1 Department of Food Safety, Nutrition and Veterinary Public Health, Istituto Superiore di Sanità, Viale Regina Elena, 299, 00161 Rome, Italy; chelli.eleonora@gmail.com (E.C.); elisabetta.suffredini@iss.it (E.S.); dario.demedici@iss.it (D.D.M.); gaia.scavia@iss.it (G.S.S.); 2 Unit of Food Biotechnology, Istituto Zooprofilattico Sperimentale del Lazio e della Toscana “M. Aleandri”, 00178 Rome, Italy; paola.desantis@izslt.it (P.D.S.); biancamaria.varcasia@izslt.it (B.M.V.); 3 Unit of Viral Diagnoses, Istituto Zooprofilattico Sperimentale della Sicilia, 90129 Palermo, Italy; santina.dibella@izssicilia.it (S.D.B.); francescagucciardi30@gmail.com (F.G.); annalisa.guercio@izssicilia.it (A.G.); giuseppa.purpari@izssicilia.it (G.P.); 4 Ministry of Health, Directorate-General for Prevention, 00144 Rome, Italy; stefania.damato@sanita.it; 5 Department of Veterinary Medical Sciences, University of Bologna, 40064 Ozzano dell’Emilia (BO), Italy; fabio.ostanello@unibo.it; 6 Veterinary Service, Department of prevention, Local Health Unit Az. USL Roma 2, 00157 Roma, Italy; vitantonio.perrone@aslroma2.it; 7 Regional Department for Health Activities and Epidemiological Observatory of the Sicilian Region, 90145 Palermo, Italy; p.schembri@regione.sicilia.it

**Keywords:** hepatitis E virus, pigs, zoonosis, genotype 3, slaughterhouse, abattoir, Italy

## Abstract

**Simple Summary:**

Hepatitis E is now recognized as an emerging zoonotic disease in Europe caused by an RNA virus (HEV) and foodborne is the main route of transmission. Human cases have been linked to the consumption of contaminated pig liver sausages, raw venison, or undercooked wild boar meat. The zoonotic genotype HEV-3 is widespread in pigs at farm level but little information is available on the occurrence of HEV-positive pigs at the slaughterhouse. The aim of this study was to investigate the prevalence of HEV-positive pigs during slaughtering, to understand which biological samples (feces and organs) were more frequently HEV positive. Our results showed that pigs positive for HEV can be slaughtered and that the percentage of positive animals depends on the age of animals. The other main result is the presence of the virus in the plasma of animals, which may contribute to the contamination of meat (muscle). Nevertheless, muscles are rarely contaminated by HEV-RNA compared to liver, which is the organ of replication.

**Abstract:**

In Europe, foodborne transmission has been clearly associated to sporadic cases and small clusters of hepatitis E in humans linked to the consumption of contaminated pig liver sausages, raw venison, or undercooked wild boar meat. In Europe, zoonotic HEV-genotype 3 strains are widespread in pig farms but little information is available on the prevalence of HEV positive pigs at slaughterhouse. In the present study, the prevalence of HEV-RNA positive pigs was assessed on 585 animals from 4 abattoirs located across Italy. Twenty-one pigs (3.6%) tested positive for HEV in either feces or liver by real-time RT-PCR. In these 21 pigs, eight diaphragm muscles resulted positive for HEV-RNA. Among animals collected in one abattoir, 4 out of 91 plasma tested positive for HEV-RNA. ELISA tests for the detection of total antibodies against HEV showed a high seroprevalence (76.8%), confirming the frequent exposure of pigs to the virus. The phylogenetic analyses conducted on sequences of both *ORF1* and *ORF2* fragments, shows the circulation of HEV-3c and of a novel unclassified subtype. This study provides information on HEV occurrence in pigs at the slaughterhouse, confirming that muscles are rarely contaminated by HEV-RNA compared to liver, which is the most frequently positive for HEV.

## 1. Introduction

Hepatitis E is an acute disease affecting humans, which is widespread globally [1]. The causative agent, hepatitis E virus (HEV) is a small virus with a single strand RNA genome, and strains belonging to the species *Orthohepevirus A* are responsible for hepatitis in humans [2]. Two main transmission pathways characterize hepatitis E epidemiology. In low-income countries, HEV infection is usually associated with large waterborne outbreaks [3], while in industrialized countries it is considered a foodborne infection, mainly causing sporadic cases and small outbreaks [3,4]. The two epidemiological patterns are linked to different viral genotypes: HEV-1 and HEV-2 for the former and HEV-3 and HEV-4 for the latter. While HEV-1 and HEV-2 only infect humans, HEV-3 and HEV-4 are considered zoonotic agents with domestic and wild pigs being the main reservoirs. HEV-3 is the most frequently detected genotype and circulates worldwide, while HEV-4 is considered endemic in Asia [5] and has only been detected twice in Europe in pigs and in sporadic human cases [6,7]. In Europe, consumption of raw or undercooked HEV contaminated sausages containing pig liver and wild boar meat has been associated with the transmission of HEV to humans [8]. The involvement of such foodstuff was supported by both virological evidence (i.e., the detection of the same viral sequence in patients and leftovers food) and analytical epidemiology studies indicating that the consumption of foods containing pork liver were associated with an increased risk of either HEV-3 or HEV-4 infection [9]. In EU countries, the pig meat sector is the main production and the type of meat most frequently consumed [10]. This data clearly explains the importance of pork meat and products thereof for the food chain.

Pigs are susceptible to HEV infection after the loss of maternal immunity [11], at the age of 3–4 months [12]. After exposure to the HEV, by the fecal–oral route, the infection is followed by viremia, production of IgM, replication of the virus in the liver, shedding of HEV in feces followed by the IgG response [13]. The duration of each stage is variable and depends on several factors, such as the presence of coinfections, passive immunity and the type of farming [14,15]. To date, it is not clear, how long the protective immunity lasts and if animals can be reinfected later on in their life. Several studies reported a wide circulation of HEV on farms in Europe and in animals older than five months [12], supporting the hypotheses that also infected animals could be sent for slaughter, therefore being a source of food contamination and foodborne exposure of humans. Several studies conducted in pigs at slaughtering allowed to detect the presence of HEV in liver, in feces and more rarely in muscles [16,17,18].

HEV-viremic pigs at the slaughterhouse may contaminate muscles through inadequate bleeding. However, occurrence of viremia in pigs at the slaughterhouse was rarely investigated and its prevalence was 0.9% in China [19], 5.7% in the UK, 6.3% in the USA and up to 44.4% in Scotland [20,21,22].

In France, a sampling study on 1134 animals yielded a HEV prevalence of 2.8% in liver samples and no muscles specimen resulted in being positive [16]. Similarly, in a study conducted in Spain, 7 out of 45 pigs were HEV positive in the liver but no muscle samples were HEV positive [23]. The probability to detect HEV in the animal liver depends on age, rising from 0.25% in livers from six month old animals to 2.5% in younger animals (three months old) during slaughtering [24]. In Italy, studies investigating the occurrence of HEV in pigs at the slaughterhouse reported from 0 to 5% of HEV-RNA positive livers [25,26], most of the studies having been conducted in Northern Italy.

The virus circulates widely in Italy in the human population as proved by several studies on the detection of antibodies against HEV in blood donors [27,28,29,30]. At the national level, the mean seroprevalence in blood donors is 8.7% with a highly variable range depending on geographical area investigated. In some regions of the Central Italy (Abruzzo and Sardinia) the mean seroprevalence grows above 15%. Both regions are not the main areas of pig farming, mostly located in Northern Italy, where the mean seroprevalence in humans varies between 5 and 10%. The observed high seroprevalence in these hyperendemic regions in Central Italy was deemed to be linked to eating habits such as consumption of raw liver sausages [27,28,29].

In this study, we aimed to investigate the presence of HEV-RNA in matrices, including the liver, of pigs from four slaughterhouses located in Northern, Central and Southern Italy. Detection of antibodies against HEV was also investigated to evaluate to which extent adult pigs were exposed to the virus.

## 2. Materials and Methods

### 2.1. Slaughterhouse and Sampling

Pig production in Italy is characteristically oriented towards typical meat products. In 2019, the Italian pig population was more than 8.5 million heads. Pig farms numbered around 32,000 in 2019 and over 88% were concentrated in Northern Italy [31]. The Italian pig sector is primarily focused on the production of heavy pigs, used to provide thighs for dry-cured ham. Out of the total 1,148,000 pigs slaughtered in Italy in 2019, 93.2% (10,704,255) had a slaughter live weight higher than 160 kg and 9–10 months of age [31].

From October 2017 to July 2019, in the framework of the national project “Hepatitis E, an emerging problem in food safety: ‘One Health’ approach for risk assessment” (CCM 2016 program) financed by the Italian Ministry of Health, 585 pigs were sampled at different points of the slaughtering line in four abattoirs located across Italy (one in Northern Italy, abattoir A; two in Central, B and C, and one in Southern Italy, D). The four abattoirs were chosen to be representative of the geographical distribution of the pig production chain in Italy in slaughterhouse A, about 700 pigs/day were slaughtered exclusively from intensive pig farms located in Northern Italy (area with the highest density of pigs in Italy). In abattoirs B and C, 100–200 pigs/day were slaughtered from farms located in Northern Italy (about 30%) and Central Italy (70%). In slaughterhouse D, about 50 animals/day were slaughtered, coming from farms in Southern Italy (area with low density of pigs). Animals imported from other EU countries were only sampled in the abattoir D.

Overall, 70 batches (groups of animals bred in the same farm) were examined. At least 2 pigs/batch were sampled (minimum 2, maximum 70 and median 2). Paired sampling of liver, feces and diaphragmatic muscle was performed whenever possible for each animal.

During bleeding, 335 individual blood samples were collected using sterile plastic bags, whose content was immediately transferred into 50 mL sterile plastic tubes. During evisceration, feces (about 50 g) were collected from large intestines and also stored in sterile plastic tubes. Liver and diaphragmatic muscle were also collected (1 cm^3^ from 3 different locations) during veterinary inspections using disposable blades (Table 1). After transport to the laboratory in a cool box, all samples were stored at −80 °C, except for blood that after 12 h at 4 °C was centrifuged at high speed (3000× *g*) and the sera recovered and stored in small aliquots at −20 °C.

### 2.2. Nucleic Acid Extractions from Fecal Samples

A total of 200 mg of feces were suspended in phosphate-buffered saline (PBS) to a final 10% *w/v* ratio, were vortexed for 60 s and centrifuged at 3000× *g* for 15 min. One hundred and fifty microliters of supernatant were immediately used for nucleic acid isolation or stored at −70 °C. Nucleic acid was extracted using the QIAamp Viral RNA Mini Kit (QIAGEN, Hilden, Germany) following the manufacturer’s instructions. Final elution was performed with 100 μL of water and samples were stored at −80 °C.

### 2.3. Nucleic Acid Extractions from Diaphragmatic Muscle Samples

Sample preparation was done according to Szabo et al. [32] with slight modifications. Muscle was grounded and 5.0 ± 0.2 g were transferred to a stomacher bag and spiked with 10 μL of a suspension of Mengovirus (strain MC_0_, 1.6 × 10^5^ TDCI_50_/mL) or murine norovirus (1.5 × 10^5^ TDCI_50_/mL), which were used as sample process controls. Seven milliliters of TriReagent Solution (ThermoFisher Scientific, Frederick, MD, USA) were added and samples were homogenized at a maximum speed for 2 min. The liquid phase was clarified by centrifugation (10,000× *g*, 20 min, 4 °C), recovered and 1.4 mL of chloroform was added. After 15 min at room temperature with gentle shaking, the supernatant was recovered after centrifugation 10,000× *g* for 15 min a 4 °C and measured. Two hundred μL were subsequently used for RNA extraction using an automated extractor (NucliSens MiniMag, bioMerieux, Marcy l’Etoile, France) according to the manufacturer’s protocol. Total RNA was eluted in 100 μL and stored at −80 °C until testing.

### 2.4. Nucleic Acid Extractions from Liver Samples

Two hundred milligrams of samples, cut from the inner part of the liver, were spiked with the process control virus as above described and were homogenized with 50 mg zirconia beads using a mechanical disruptor (Tissue Lyser, QIAGEN, Hilden, Germany) for three runs of 2 min at 46 oscillations s^−1^ or grounded with cold ice. After centrifugation at 10,000× *g* for 15 min at 4 °C the recovered supernatant was subjected to RNA extraction using the Qiamp Viral kit (QIAGEN, Hilden, Germany) following the manufacturer’s instruction and eluted in 100 μL. RNA was stored at −80 °C until testing.

### 2.5. Nucleic Acid Extractions from Plasma

Total RNA was extracted from plasma using the QiAmp Viral RNA mini kit (QIAGEN, Hilden, Germany), following the manufacturer’s instruction. RNA was eluted and stored as described.

### 2.6. Nucleic Acid Extraction Efficiency

The RNA of mengovirus or murine norovirus process controls were detected by real-time RT-qPCR as previously described [33,34]. The extraction efficiency was estimated by the comparative cycle threshold (*Ct*) method [35] between mengovirus or murine norovirus (MuNoV) detected in the spiked sample and mengovirus or MuNoV used for spiking. RNAs from samples negative for HEV and displaying <1% of recovery were extracted again.

### 2.7. HEV Real-Time RT-qPCR

For HEV detection, a 5 µL aliquot of sample RNA was analyzed using the RNA UltraSense™ One-Step qRT-PCR System (Thermofisher Scientific, Frederick, MD, USA), 1.25 µL of the RNA Ultrasense enzyme mix and the following concentrations for primers and probe: 500 nM for forward primer JVHEVF (5′-GGTGGTTTCTGGGGTGAC-3′), 900 nM for reverse primer JVHEVR (5′-AGGGGTTGGTTGGATGAA-3′) and 250 nM for probe JVHEVP-MGB (5′-FAM-TGATTCTCAGCCCTTCGC-MGB-3′) [36,37]. Reverse transcription was done at 50 °C for 60 min, and was followed by inactivation for 5 min at 95 °C and by 45 cycles of 15 s at 95 °C, 1 min at 60 °C and 1 min at 65 °C.

For positive samples, quantitative analysis was performed using the same real-time RT-PCR with the procedure (number of replicates, standard curves construction, acceptability criteria, etc.) described in Di Pasquale et al. [38].

### 2.8. Sequencing and Subtyping

Samples HEV-RNA positive by real-time RT-PCR were subjected to two nested RT-PCRs amplifying a 348 bp genome fragment in the *ORF2* [39,40] and a 287 bp within the methyltransferase (*ORF1*) [41]. The DNA amplicons of expected size were sequenced (Eurofins Genomics, Hamburg, Germany) and sequences after editing were uploaded into the NCBI database (https://www.ncbi.nlm.nih.gov) [42] under the accession numbers: MW136484-MW136500. The sequences were analyzed to establish the genotypes and the subtypes by BLAST analysis and using the HEV Typing Tool 0.1 (http://www.rivm.nl/mpf/typingtool/hev/) [43].

A maximum likelihood (ML) phylogenetic tree was constructed with the Tamura-Nei parameter model and Gamma distribution by the MEGA 7 software (http://www.megasoftware.net). The sequence dataset used to build the tree and determine the subtype includes the reference strains of HEV-3 indicated by Smith et al. [44] and HEV strains detected in Europe, including Italy, available on the NCBI database.

### 2.9. Meat Juice Preparation

To perform serological assays, the muscle samples were put at −20 °C overnight and were thawed at room temperature before use. The samples were centrifuged 8000× *g* for 5 min to collect meat juice. The obtained volume of meat juice differed from sample to sample and ranged from 20 to 1000 µL.

### 2.10. Antibodies Detection

Total antibodies against HEV in pig sera and pig meat juice were detected by commercial ELISA test (HEV-Ab, Wantai Biopharmaceutical Inc., Beijing, China) following the manufacturer’s instructions.

### 2.11. Statistical Analysis

Animals were sampled during several visits (2017–2019) to the plants. Information on age, weight and farm of origin of animals (North, Centre, South) was collected. Based on the available data, HEV point prevalence and 95% confidence intervals (95% CI) estimates were stratified according to the slaughterhouse, geographical location of the farm of origin and weight at slaughter.

Pigs were considered positive when HEV-RNA was detected in the liver and/or in fecal samples examined. Correlations between HEV detection and animal age, presence of anti-HEV antibodies, geographical location of the farm of pig origin and geographic location of the slaughterhouse were evaluated using a univariable chi-square test. For the analysis, animals were categorized in two groups: lightweight pigs, intended for the production of fresh meat (87 animals, mean age: 4.5 months; minimum 1 and maximum 7) and heavy pigs (intended for provide thighs for dry-cured ham or meat for other cured-meat product) (498 animals, mean age: 9 months; minimum 9 and maximum 10). Confidence intervals were calculated by binomial (Clopper–Pearson) “exact” method based on the β distribution.

All statistical analyses were performed using the software SPSS 26.0.0 (SPSS Inc., Chicago, IL, USA).

## 3. Results

A total of 585 livers were sampled from pigs and, when possible (16 fecal samples were not collected), paired feces were also collected. HEV-RNA detection was obtained in 12 animals in the liver (2.1%; 95%CI: 1.1–3.6) and 11 in the feces (1.9%; 95%CI: 1.0–3.4); among these, two animals were positive in both types of samples (0.35%; 95%CI: 0.0–1.3). Overall, 21 animals out of 585 (3.6%; 95%CI: 2.2–5.4) were positive in at least one of the two tested sample types. Diaphragm muscles were also analyzed for those animals testing positive in either liver or feces. Overall, 8 animals were also positive in the diaphragm, and two animals were positive in all the three matrices.

Viral concentration in liver ranged from 1.5 × 10^6^ to 2.7 × 10^8^ genome equivalents (GE)/g with a median concentration of 6.7 × 10^6^ GE/g. For diaphragmatic samples, because below the quantification limit, no quantitation could be obtained. Fecal samples (no. = 10) showed the largest variation of viral concentration ranging from values below the quantification limit (1.12 GE/µL of RNA=7.5 × 10^3^ GE/g) to 4.0 × 10^7^ GE/g, with a median value of 1.2 × 10^4^ GE/g.

The comparison of HEV prevalence among the animals grouped according to the area of slaughterhouse (Northern, Central and Southern Italy) showed a statistically significant difference (Table 2) (*p* < 0.001), in one abattoir no positive animals were detected. This statistically significant difference (Table 2) (*p* < 0.001) was also confirmed by grouping based on the geographical origin (i.e., farm origin) of the animals. In the latter analyses, the prevalence was similar in Northern and Central Italy, 2/228 (0.9%; 95%CI: 0.1–3.1) and 2/254 (0.8%; 95%CI: 0.1–2.8) respectively (Table 2) and rose to 15.4% (95%CI: 8.7–24.5; 14/91) in animals farmed in Southern Italy. By comparing the prevalence for the age groups, a significant statistical difference (*p* < 0.001) was also observed, with a higher prevalence in lightweight pigs (10/87; 11.5%; 95%CI: 5.7–20.1) than in heavy pigs (11/498; 2.2%; 95%CI: 1.1–3.9) (Table 2). No positive animals (in either fecal or liver samples) were detected in animals younger than five months (25 animals). A few pigs (12) were sampled among batches imported from other European countries and slaughtered in Southern Italy. Three out of 12 animals (25%; 95%CI: 5.5–57.2) resulted in being positive for HEV-RNA in feces, all originated from the same batch (Table 2).

A total of 335 sera and 74 meat juice samples were collected and analyzed to detect whole antibodies against HEV. Overall, 76.8% (95%CI: 72.4–80.8; 314/409) of sera and meat juice were positive for the presence of anti-HEV antibodies. No significant difference (*p* = 0.08) in seroprevalence was observed based on age groups, with seroprevalence being somewhat higher for lightweight pigs (73/87; 83.9%; 95%CI: 74.5–90.9) (Table 3). A statistical difference (*p* = 0.015) was observed between seroprevalence detected in animals raised in the three Italian areas (North, South and Center) being higher in animals farmed and slaughtered in the South (Table 3).

Overall, seventeen animals were both seropositive and positive for HEV-RNA in liver or feces or muscles, but the difference between the presence of antibodies and HEV-RNA was not significant (*p* = 0.17).

Among animals farmed in Italy and collected in the abattoir in Southern Italy (slaughterhouse D), 91 animals were randomly selected and testing of paired liver, feces and additionally plasma was performed. Overall, four plasma were positive (4.4%) of which one was negative for all other matrices (liver and feces), one was also positive in the diaphragmatic muscle and two also in feces. Quantitative real-time RT-PCR was conducted and viral concentration in plasma samples ranged between 4.4 × 10^3^ and 2.5 × 10^4^ GE/mL, with a median value of 7.3 × 10^3^ GE/mL. Eighty-one out of 91 pigs had also antibodies against HEV (87.1%) and 2 had both antibodies and HEV-RNA.

Correlation between the presence of antibodies (No. 409 tested animals) and detection of HEV-RNA (No. 585 tested animals) was evaluated (Table 4).

**Figure 1:**
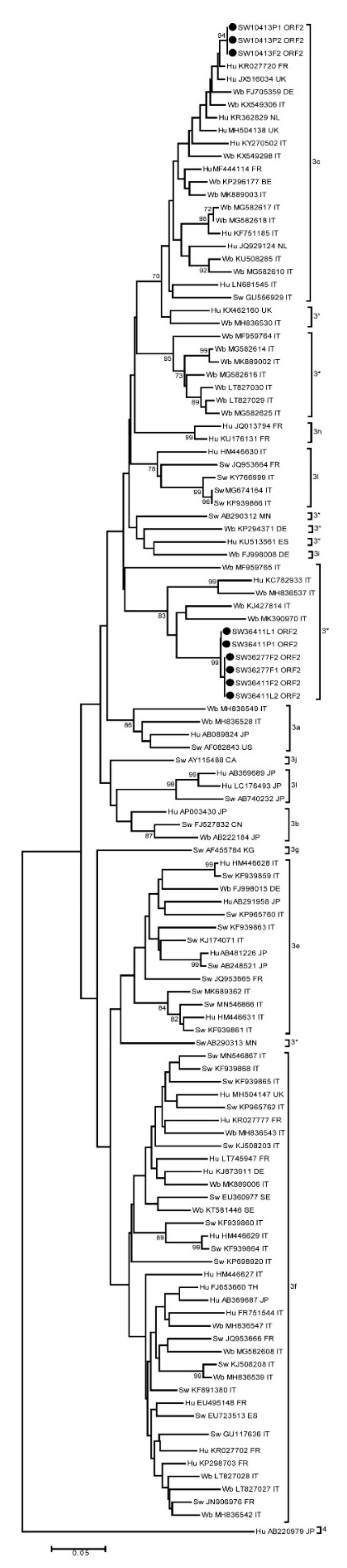
Maximum likelihood phylogenetic tree based on 328 nt fragment within the HEV *ORF2* region of HEV-3 strains from human, pig and wild boar including HEV-3 reference strains. The HEV-4 strain was used as an outgroup. The Italian strains sequenced in this study are indicated by a black circle. Bootstraps values > 70 are indicated at their respective nodes. Representative porcine, human, wild boar and monkey strains are included. Each entry includes host (Hu: human, Sw: swine and Wb: wild boar), accession number and countries origin of strains.

Nine HEV-RNA positive diagnostic PCR fragments of both *ORF1* and *ORF2* were amplified and subjected to sequencing. All sequences belonged to animals slaughtered in Southern Italy, three from pigs imported from the EU country and the other six were from animals farmed in Southern Italy. Three animals were from the same farm and for three of them sequences from different sample types (i.e., liver, feces, etc.) were obtained and were identical to each other (Figure 1). Based on sequence analyses of the *ORF2* genome fragments, two clusters of viral strains were observed. The first one, including six sequence strains, was an unclassified 3* as the sequence was not classifiable in any of the subtypes defined so far. The six sequence strains were related to both human (Acc. No. KC782933) and wild boar (Acc. No. KJ427814) HEV strains detected in Italy in the previous studies years ago; the second cluster with three sequence strains belonged to HEV-3c, and was related to human cases detected in the UK (Acc. No. JX516034) and in France (Acc. No. KR027720). Similar results were also confirmed by analyses and alignment of *ORF1* genome fragment sequences. 

## 4. Discussion

This study aimed to evaluate the prevalence of HEV positive pigs entering the food chain in Italy, by detecting infected animals with viral RNA in feces and/or liver. Twelve animals tested positive for HEV in the liver only (2.1%), 11 in the feces only (1.9%) and two of them in both matrices. Comparison of these results with previous studies is difficult, because a different number of animals and/or abattoirs were analyzed. Nevertheless, the point prevalence value estimated in this study was lower when compared to previous Italian studies (6.06% liver and 7.3% of feces) [18,45] and it is noteworthy that other studies in Italy also reported absence of HEV in pig livers sampled in different abattoirs [25,46]. However, if compared with recent studies conducted in other European countries (France, Slovenia and Spain), results obtained were comparable (2.1–2.8% in liver) [16,24,26]. Similarly, taking into consideration the number of pigs positive in feces, the overall prevalence detected was 1.9%, which is below the prevalence observed in pigs on farms in Italy (prevalence at the farm level ranging from 24.8 to 100%) [25,47,48] and in pigs at the slaughterhouses in other EU countries [23,26,49].

Although the observed differences may reveal true epidemiological differences, they could also reflect differences in the methodology used for samplings, i.e., number and age of animals or abattoirs analyzed and/or differences in methods used for HEV detection or it could represent geographical differences, making comparisons among studies difficult. Indeed, previous studies conducted in Italy analyzed prevalently animals bred and slaughtered in Northern Italy [18]. In this study, a higher number of animals and abattoirs were enrolled across the whole country compared to previous studies conducted in Italy [18,26].

Another hypothesis to justify the overall low prevalence observed could be linked to the age of animals. The detected prevalence was significantly higher (11.5%) in lightweight pigs (mean age: 4.5 months) and lower (2.2%) in heavy pigs (mean age: 9 months). The correlation of HEV-RNA occurrence and the age of animals, with a significant lower prevalence in older animals, has been frequently described [15,24]. In this study, HEV-RNA positive animals were detected in all but one abattoir, which was located in Northern Italy where only heavy fatteners locally bred are slaughtered, confirming a reduced probability of detecting HEV in older pigs. In Italy, the majority of pigs entering the food chain are heavy weight (mean 165 kg) for cured meat production and are slaughtered at 9–10 months of age. This category includes fewer and almost no HEV positive animals (2.2%), the age favoring the clearance of HEV infection, suggesting that the risk of being a vehicle for HEV infection is low.

The type of farm of animal origin could have also influenced the exposure of animals to the virus and the probability to be infected [50]. Unfortunately, information about the type of farms, where pigs enrolled in the study were raised and bred, was not available, but future studies will aim to evaluate possible correlations between the risk to be infected and other parameters correlated to pig farming. A data gap in this study is the lack of information on farms of origin of the pigs analyzed and the sampling of a representative number of animals per farm that hampers the calculation of prevalence per farm. Unfortunately, only four abattoirs were sampled but those enrolled in the study well represents the differences of Italian pig productions: the abattoir located in Northern Italy (where most pig producers are located) was the biggest with 700 heads slaughtered per day, followed by Central Italy (100–200 heads per day) and the South where 50 pigs were slaughtered per day.

The likelihood that meat is contaminated by HEV is linked to the probability of slaughtering viremic animals that may determine the presence of the virus in muscles. In our study, the presence of HEV-RNA in plasma was investigated in 91 pigs, with 4.4% of the pigs testing positive. This is the first study in Italy to analyze viremia, which has rarely been investigated thus far. Studies conducted so far have shown a similar percentage of 5.7% in the UK [20] and 6.3% in the USA [21]. Viremia is transient and more difficult to detect but represents a major risk of muscle contamination linked to residual blood after bleeding. However, in our study only one animal was found HEV-RNA positive in paired diaphragmatic muscle and plasma.

In this study, we also investigated the presence of anti-HEV antibodies. Results showed that pigs were frequently exposed to the virus, as confirmed by a seroprevalence higher than 56.7% in animals from all abattoirs, varying between 56.7 and 100%, and correlated to the geographical origin of animals. The meat juice was used for antibody detection in pigs from one out of the two abattoirs in Central Italy. The use of meat juice should not significantly influence the result, since its use has been proven as equivalent to sera for antibody detection [51,52]. In our study 9 out of 12 animals positive in the liver for HEV were also seropositive, which could be due to the timing of the infection since the presence of antibodies against HEV rises after the stage of replication in liver [53,54,55] or could be due to a lack of protection in the presence of antibodies. The study conducted in the UK on viremia and antibodies in pigs at slaughterhouse produced similar results: most HEV-RNA positive pigs were seropositive and half of them also had detectable HEV IgM [20]. We did not know if animals seropositive in our study had IgM, which would have indicated an acute/recent infection, but the presence of antibodies was not sufficient to exclude the presence HEV infection.

The highest viral load, measured as genome equivalents, was detected in the liver with a median value of 6.7 × 10^6^ GE/g, which is expected considering that liver is the organ of viral replication. A significantly lower titer was detected in plasma 7.3 × 10^3^ GE/mL and was below the limit of quantification in muscles, suggesting that even in the phase of viremia the amount of virus circulating is limited. This confirms that liver may be the riskiest ingredient in pork products when consumed raw.

The HEV infectious dose in humans is unknown, and in vivo experiments in pigs showed a dose/dependent infection with at least 10^6^ GE needed to observe seroconversion in pigs inoculated intravenously [11]. Nevertheless, pigs inoculated with pig patè containing HEV-3-infected liver (2.24 × 10^7^ GE/g) succeeded, confirming the presence of infectious virus in the liver with a viral load comparable to our result [56]. Ten pigs were shedding the virus in feces with large viral loads variations. It is difficult to provide the reason for this result. Viral load variability in fecal samples may represent a different stage of the infection, but there are currently no data available that correlate HEV fecal viral load in shedding pigs with the progression of infection. It is noteworthy that in five pigs the viral load measured in terms of GE was below 1 GE/µL, reducing the probability of transmission among animals, considering that HEV in pigs is transmitted by the fecal oral route [57].

Due to the low titer for some of the positive samples we only obtained sequences from seven animals (with multiple sequences from the same animal). Although short genome fragments were analyzed (both *ORF2* and *ORF1*), sequence results confirmed the presence of only a single HEV strain in each animal and in animals from the same farm. Most of the strains belong to an unclassified subtype of HEV-3 [58], which includes strains only detected in Italy in wild boar and in a sporadic case in Northern Italy in 2012 [59,60]. We cannot exclude the possibility that this subtype only circulates in Italy since it has not been identified abroad so far. Most interestingly, the sequence strains detected from three pigs imported from other EU countries to Italy for slaughtering belong to HEV-3c. The sequence of strains identified in this study is strictly related to sequence strains detected in humans in the UK and in France [61]. This subtype is very common in Europe in both pigs and humans [62]. In Italy, the HEV-3c is rare, it has only been reported once in pigs [45]. It has also been rarely reported in humans where the subtype 3e is predominant [14]. In Italy, 30% of pigs (1,342,000 heads) are imported from other European countries for slaughtering [31]. Although this percentage is limited compared to other EU countries it could determine the introduction of new subtypes from abroad.

## 5. Conclusions

The results obtained in this study corroborated the wide circulation of the zoonotic HEV-3 in Italian pigs, revealed by the high percentage of detection of anti-HEV antibodies. Nevertheless, the probability of HEV RNA-positive pigs at slaughter was low, as the probable result of an infection clearance with the age of the animals. Heavy pigs were slaughtered later than light pigs. In our study, the latter resulted in the group being most frequently positive for HEV-RNA. This result suggests that differences in the percentage of positive pigs could exist among pigs raised up and bred in different European countries also depending on the age of slaughter and that surveillance studies on HEV circulation at farm are needed to evaluate differences among countries and factors (i.e., age, type of farm and biosecurity measures) influencing the occurrence of HEV infections. The transmission of the zoonotic HEV-3 to humans can be prevented by pork being cooked properly or with procedures to inactivate the virus in raw pork products, such as sausages. Since it is not clear yet for how long the virus survives in pork meat products, it is important to prevent the introduction of animals with active viral replication at the slaughterhouse, by implementing biosecurity measures on farms and by the continuously monitoring of HEV in fattening pigs close to slaughter.

## Figures and Tables

**Table 1:** Scheme of sampling, summarize matrices used and techniques applied for the analyses.

Samples	Diagnostic Test Used	Total Examined Samples
Liver	Real-time RT-qPCR	585
Feces	Real-time RT-qPCR	569
Plasma	Real-time RT-qPCR	91
Muscle	Real-time RT-qPCR	21 *
Sera	ELISA	335
Meat juice	ELISA	74

* Only diaphragm from pigs positive for HEV-RNA in the liver or feces.

**Table 2:** Comparative analyses of percentage of pig positive for HEV-RNA in liver and/or feces.

Variables	Categories	Total Pigs	No. Pigs HEV-RNA Positive			*P*
			No.	(%)	95%CI	
Geographic area of the slaughterhouse	Northern Italy (A)	120	0	(0.0)	[0.0–3.0]	<0.001
	Central Italy (B and C)	362	4	(1.1)	[0.3–2.8]	
	Southern Italy (D)	103	17	(16.5)	[9.9–25.1]	
Geographical origin of pigs slaughtered	Northern Italy	228	2	(0.9)	[0.1–3.1]	<0.001
	Central Italy	254	2	(0.8)	[0.1–2.8]	
	Southern Italy	91	14	(15.4)	[8.7–24.5]	
	Other EU countries	12	3	(25.0)	[5.5–57.2]	
Age class	lightweight pigs (4.5 months ^1^)	87	10	(11.5)	[5.6–20.1]	<0.001
	heavy pigs (9 months ^1^)	498	11	(2.2)	[1.1–3.9]	
Total		585	21	(3.6)	[2.2–5.4]	

^1^ mean age.

**Table 3:** Results of anti-HEV antibodies detection in pigs grouped for slaughterhouse, geographical origin and age classes.

Variables	Category	Total Pigs	No. Positive Pigs for Anti-HEV Antibodies			*p*
			No.	%	95%CI	
Geographic area of the slaughterhouse	Northern Italy (A)	120	68	(56.7)	[47.3–65.7]	<0.001
	Central Italy (B)	115	115	(100)	[96.8–100]	
	Central Italy (C) ^1^	73	44	(60.3)	[48.1–71.6]	
	Southern Italy (D)	101	87	(86.1)	[77.8–92.2]	
Geographical origin of pigs slaughtered	Northern Italy	210	149	(71.0)	[64.3–77.0]	0.023
	Central Italy	98	78	(79.6)	[70.3–87.1]	(0.015) ^3^
	Southern Italy	91	78	(85.7)	[76.8–92.2]	
	Other EU countries	10	9	(90.0)	[55.5–99.8]	
Age class	lightweight pigs (4.5 months) ^2^	87	73	(83.9)	[74.5–90.9]	0.08
	heavy pigs (9 months) ^2^	322	241	(74.8)	[69.3–79.5]	
Total		409	314	(76.8)	[72.4–80.8]	

^1^ In slaughterhouse C the meat juice was examined; ^2^ mean age and ^3^ when only animals reared in Italy are considered.

**Table 4:** Summary of results about the presence of antibodies anti-HEV and HEV-RNA in the same animal.

	Presence of HEV-RNA			
Anti-HEV Antibodies	Liver	Feces	Muscle	No. of Animals
Positive (No. 17)	+ ^1^	+	+	2
	+	− ^2^	+	5
	+	−	−	2
	−	+	−	8
Negative (No. 3)	+	−	+	1
	+	−	−	2
Not tested for antibodies (No. 1)	−	+	−	1

^1^ + positive; ^2^ − negative

## Data Availability

The sequence data presented in this study are openly available in NCBI database (https://www.ncbi.nlm.nih.gov) under the accession numbers: MW136484-MW136500.
